# Bacteria and Their Antibiotic Resistance Profiles in Ambient Air in Accra, Ghana, February 2020: A Cross-Sectional Study

**DOI:** 10.3390/tropicalmed6030110

**Published:** 2021-06-25

**Authors:** Godfred Saviour Kudjo Azaglo, Mohammed Khogali, Katrina Hann, John Alexis Pwamang, Emmanuel Appoh, Ebenezer Appah-Sampong, Meldon Ansah-Koi Agyarkwa, Carl Fiati, Jewel Kudjawu, George Kwesi Hedidor, Amos Akumwena, Collins Timire, Hannock Tweya, Japheth A. Opintan, Anthony D. Harries

**Affiliations:** 1 Environmental Protection Agency, Ministries Post Office, P.O. Box MB 326, Accra, Ghana; john.pwamang@epa.gov.gh (J.A.P.); eeappoh@yahoo.com (E.A.); kwabenaappahsampong@yahoo.com (E.A.-S.); meldonagyarkwa6@gmail.com (M.A.-K.A.); uccdfiati@gmail.com (C.F.); jkudjawu@gmail.com (J.K.); 2 Special Programme for Research & Training in Tropical Diseases (TDR), World Health Organization (WHO), 1211 Geneva, Switzerland; khogalim@who.int; 3 Sustainable Health Systems, Lumley Road, Freetown, Sierra Leone; hann.katrina@gmail.com; 4 World Health Organization Country Office, P.O. Box MB 142, Roman Ridge, Accra, Ghana; hedidorg@who.int; 5 Department of Medical Microbiology, University of Ghana Medical School, Accra, Ghana; aakumwena@ug.edu.gh (A.A.); jaopintan@ug.edu.gh (J.A.O.); 6 Ministry of Health, P.O. Box CY 1122, Harare, Zimbabwe; collins.timire@theunion.org; 7 International Union against Tuberculosis and Lung Disease, 75006 Paris, France; adharries@theunion.org; 8 The Lighthouse Trust, P.O. Box 106, Lilongwe, Malawi; hmwtwea@yahoo.co.uk; 9 London School of Hygiene and Tropical Medicine, London WC1E 7HT, UK

**Keywords:** ambient air, particulate matter, pathogenic bacteria, antibiotic resistance, operational research, SORT IT, antimicrobial (AMR) resistance surveillance, air pollution, environment, Ghana Environmental Protection Agency (EPA)

## Abstract

Inappropriate use of antibiotics has led to the presence of antibiotic-resistant bacteria in ambient air. There is no published information about the presence and resistance profiles of bacteria in ambient air in Ghana. We evaluated the presence and antibiotic resistance profiles of selected bacterial, environmental and meteorological characteristics and airborne bacterial counts in 12 active air quality monitoring sites (seven roadside, two industrial and three residential) in Accra in February 2020. Roadside sites had the highest median temperature, relative humidity, wind speed and PM_10_ concentrations, and median airborne bacterial counts in roadside sites (115,000 CFU/m^3^) were higher compared with industrial (35,150 CFU/m^3^) and residential sites (1210 CFU/m^3^). *Bacillus* species were isolated in all samples and none were antibiotic resistant. There were, however, *Pseudomonas aeruginosa*, *Escherichia coli*, *Pseudomonas species*, non-hemolytic *Streptococci*, *Coliforms* and *Staphylococci* species, of which six (50%) showed mono-resistance or multidrug resistance to four antibiotics (penicillin, ampicillin, ciprofloxacin and ceftriaxone). There was a positive correlation between PM_10_ concentrations and airborne bacterial counts (r_s_ = 0.72), but no correlations were found between PM_10_ concentrations and the pathogenic bacteria nor their antibiotic resistance. We call for the expansion of surveillance of ambient air to other cities of Ghana to obtain nationally representative information.

## 1. Introduction

Antibiotic resistance, the ability of bacteria to grow or survive in the presence of an antibiotic at a concentration that is usually sufficient to kill the bacteria, is a global public health threat. It undermines the ability of antibiotics to effectively prevent and treat bacterial infections [1]. The emergence of antibiotic resistance can be linked to the irrational and indiscriminate use of antibiotics in human health and in the veterinary and agriculture sectors [2]. These poor practices have led to the presence of bacteria that are resistant to antibiotics in the environment, including ambient air [3,4]. The known sources of bacteria in ambient air include dry soil, plants, animals, dead and decaying animal bodies, sludge disposal sites, wastewater treatment plants and solid waste disposal sites [3,4]. 

Exposure to these bacteria may result in a number of adverse health effects [4] as airborne bacteria can be toxic, allergenic and/or infectious [5]. The adverse effects of airborne bacteria on human health depend on the type of bacteria and their concentrations in inhaled air. On average, humans inhale about 14 m^3^ of air per day, which may contain various types of bacteria [6]. The World Health Organization (WHO) lists 12 priority bacteria that can affect human health through inhaled ambient air [7]. 

The survival of bacteria in the air is affected by meteorological factors (temperature, relative humidity and wind velocity) and air pollutants such as carbon monoxide, hydrocarbons, nitric oxide, nitrogen dioxide, sulfur dioxide and ozone [8,9]. Air pollution is commonly measured by particulate matter (PM—made up of small particles and liquid droplets containing acids, organic chemicals, metals, and soil or dust particles), which can be classified as PM_2.5_ (fine particles) and PM_10_ (coarse particles). Sources of PM are many, and include motor vehicles, industry, wood-burning stoves, bush fires and so on. Particulate pollution with both fine and coarse particles is a public health hazard that can cause or aggravate heart and lung disease. There is also evidence to link air pollution to the onset or exacerbation of respiratory infections, and it is therefore of interest to know whether increasing levels of PM are associated with higher airborne bacterial concentrations [9]. 

Information on bacterial counts and PM concentrations in ambient air is needed, not only to estimate the health risks associated with inhaled air, but also to help strengthen and formulate strategies to minimize microbial air pollution. Importantly, countries must have the tools, required technology and skills to be able to monitor such factors. Failure to do so means that countries will fall short of fully understanding environmental health risks. This monitoring is also important to measure progress towards achieving the Sustainable Development Goals (SDG), particularly SDG 3 on good health and wellbeing, SDG 11 on making cities inclusive, safe, resilient and sustainable and SDG 13 on taking urgent action to combat climate change (United Nations General Assembly 2015).

In 2005, the Environmental Protection Agency (EPA) in Accra, Ghana, launched a program to monitor ambient air quality in 15 sites. The sites are categorized as industrial, residential, commercial and roadside and are situated in urban Accra. In 2019, and in light of the emergence of antimicrobial resistance (AMR), the EPA added the monitoring of antibiotic-resistant bacteria in ambient air to the program. 

Little is currently known about the presence of bacteria and their antibiotic resistance profile in the ambient air of Accra. A study in 2018 of indoor sites in a university, which included laboratories, classrooms, toilets and libraries, found high rates of antibiotic resistance in airborne bacteria: this included both Gram-positive cocci and Gram-negative bacilli, with 100% of the bacterial isolates showing resistance to penicillin and ampicillin [10]. However, there is no published information about airborne bacteria in outside ambient air. Such information from specific sites provides an opportunity to estimate environmental health risks related to air quality, to investigate sources of the bacteria and inform intervention strategies and policies. We therefore examined ambient air for six selected bacteria that are part of the WHO priority list of human pathogens, and also included analysis of *Bacillus* species, which are usually harmless saprophytes widespread in the community, as a form of sampling and microbiological quality control. We selected four antibiotics that are commonly used (penicillin, ampicillin, ciprofloxacin and ceftriaxone) to treat bacterial infections in Ghana.

The aim of this study, therefore, was to assess ambient air characteristics and the presence and antibiotic resistance profile of seven selected bacteria (*Pseudomonas aeruginosa, Escherichia coli*, *Pseudomonas* species, *Streptococci*, *Coliforms*, *Staphylococci* species and *Bacillus* species) in the ambient air of urban Accra, Ghana, in 2020. 

Using EPA routine monitoring data for ambient air samples collected from 12 sampling sites (seven roadside, two industrial and three residential), specific objectives were to be reported per type of site, on: (i) the environmental and meteorological characteristics and airborne bacterial counts of the samples; (ii) the presence and antibiotic resistance profiles of the seven selected bacteria; and (iii) the association between the ambient air PM_10_ concentrations and airborne bacteria counts, selected bacteria and their antibiotic resistance profile. 

## 2. Materials and Methods

### 2.1. Study Design

This was a cross-sectional study using routinely collected data on ambient air quality.

### 2.2. Study Setting

#### 2.2.1. General Setting

Ghana is a country located along the Gulf of Guinea and Atlantic Ocean, in the sub-region of West Africa. It is bordered by Cote d’Ivoire to the west, Burkina Faso to the north, Togo to the east and the Gulf of Guinea and the Atlantic Ocean to the south. 

#### 2.2.2. Specific Setting

Accra is the capital city of Ghana with a population of approximately 2.27 million. Accra’s urban area covers 225.67 square kilometers (km) of land. There are four major roads in Accra and five major hospitals [11]. In the metro area, the population density is approximately 1300 people per square kilometer. There are several slum areas situated within and around the city’s boundaries with poor hygiene and sanitation. These slums are characterized by high population density and lack of basic amenities such as housing, sanitation facilities and water supply [11]. The city can be divided into two parts by the Odaw River, which is about 5.41 km long. The largest densely populated urban slum in Accra is located along the river. The area is characterized by open burning, open defecation, extraction of materials from electrical and electronic wastes and industrial and domestic waste close to the bank of the river [11]. 

##### Meteorological Characteristics

The average rainfall in Accra is about 730 mm, which normally falls within the two main rainy seasons, occurring between April to July and September to October. The mean monthly temperature ranges from 25.9 °C in August to 29.6 °C in March. Relative humidity is generally high, varying from 65% at mid-day to 95% at night. During warmer months, the windy harmattan season makes the city breezy, dry and dusty. Drainage infrastructure in Accra is inadequate and this causes perennial flooding in the city. Average wind speed in Accra ranges from 8 to 16 km/hour. Air is often trapped in pockets over the city, and an insulation effect gives rise to local increases in air temperature of several degrees. According to EPA’s Air Quality Management Plan [12], the annual mean concentrations of particulate matter in ambient air exceeded the WHO guideline value of 50 and 25 µg/m^3^ for PM_10_ and PM_2.5_, respectively. Similarly, in the same document, the PM_10_ and PM_2.5_ concentrations exceeded the recommended limits of 70 and 35 µg/m^3^, respectively, as prescribed in the Ghana Standard [11,12]. 

##### Economic Activities

The economic activities in Accra include food and beverage manufacturing, electricity, gas, water, construction, supermarkets, shopping malls, hotels, restaurants, transportation, storage and communication. Accra is a center for manufacturing, marketing and transportation. The major transportation method in Accra is by road. The transport sector relies largely on mini buses for passengers, many of which are old and lack maintenance. The transport sector contributes massively to ambient air pollution in the city. Emissions from motor vehicles are regulated by the Ghana Standard (GS 1219:2018) for Environment and Health Protection, Requirements for Motor Vehicle Emissions [13].

#### 2.2.3. EPA of Ghana

The EPA of Ghana was established in 1994. It is responsible for regulating and ensuring the implementation of the government’s policy on the environment. The Environmental Protection Agency Act, 1994 (Act 490), mandates the agency to monitor environmental pollutants and their impact on public health [14]. 

#### 2.2.4. EPA Air Quality Monitoring Program

The EPA first started monitoring ambient air quality in Accra, Ghana, in 1998 and expanded its scope in 2005. The EPA collects ambient air quality samples on a six-day routine cycle from 15 monitoring sites located in Accra. The EPA ambient air quality monitoring is regulated by the Ghana Standard (GS 1236:2019) for environmental and health protection—there are requirements for ambient air quality at point sources and for stack air emissions [15]. The 15 sites were established to accurately characterize the severity and nature of air pollution problems in Accra and to make recommendations for the development of a broad base air quality management strategy in Ghana. 

### 2.3. Study Population and Periods

The study population included ambient air samples collected from the Agency’s air quality monitoring sites in urban Accra in February 2020. Of the 15 sites monitored by the EPA, 12 were selected for this study: three sites were not included because they were not active at the time of sample collection. One air quality sample was collected from each site. The 12 selected sites included seven roadside, two industrial and three residential sites, and these are shown in the Supplementary Materials Annex S1. 

### 2.4. Data Variables and Sources of Data

Data were collected from the EPA ambient air quality database. Variables included environmental and meteorological characteristics (temperature (°C), relative humidity (RH), wind speed (km/hr), particulate matter (PM_10_) (µg/m^3^)); bacterial organisms (*Pseudomonas aeruginosa*, *Escherichia coli*, *Pseudomonas* species*,* *Streptococci*, *Bacillus* species, *Coliforms* and *Staphylococci* species); and the resistance profile of bacterial isolates to four selected antibiotics (penicillin, ampicillin, ciprofloxacin, ceftriaxone).

The specific component of the machine for measuring PM_2.5_ was not available for the study and, thus, PM_2.5_ concentrations in the ambient air could not be measured. 

### 2.5. Sampling and Testing Procedures and Data Collection

#### 2.5.1. Sampling and Testing Procedures

##### Routine Air Quality Sampling Procedure

Samples were obtained using an air sampler equipped with sterile quartz filters (47 mm in diameter; 0.45 μm Millipore). The air sampler was operated at an airflow rate of 5 L/min, which was maintained by a vacuum pump (Tactical MiniVol air sampler) and verified with a calibrator (Mass Flowmeter). The equipment for sampling air was located at an altitude of 1.5 m above ground level to prevent the collection of ground level dust. The ambient air was siphoned through the filter paper, mounted in the sampling unit and sampling was undertaken for 24 h at each site. Before sampling, the empty sampling filter cassettes were labeled with the site ID and the sampling date. The filter papers were then placed in filter cassettes using sterile forceps for sterilization in a drying oven. The sterilized quartz filter papers were weighed in a sterilized weighing room. The weighed filter papers were then transferred into a filter holder unit using sterile forceps. The empty filter cassettes were aseptically wrapped in aluminum foil and kept in the desiccator. Site identification tags were placed on the filter holder units so that the ID number of the filter mounted in the holder unit was known. 

The sterile filter holder unit and plastic bags were kept frozen at −4 °C for transportation to the site. The filter holder units were kept in a vertical position until installed in the field. After the 24 h sampling period, the post-filter paper with the holder units was removed and kept frozen at −4 °C for transporting back to the laboratory for weighing under sterilized conditions. For the particulates, the EPA uses gravimetric testing methods. 

##### Quality Assurance for Routine Air Quality Sampling Procedure

A routine air quality monitoring and assurance program encompassed all phases of ambient air sampling and data analysis. All phases included: monitoring site validation, monitoring equipment audit/calibration and procedures, sampling procedures, data validation, data reporting, precision/accuracy reporting and meteorological characteristics. 

Filter papers were kept under controlled temperatures before weighing for sampling and after sampling to avoid cross-contamination in the Environmental Quality Department laboratory of the Agency. The filter papers for microbiology analysis were kept in sterile filter cassettes, wrapped in aluminum foil, packed in zip-lock bags and kept frozen at −4 °C. The filter papers were then placed in an ice cooler and transferred to the University of Ghana Medical School, Department of Medical Microbiology, for microbiological analysis. 

##### Microbiology Procedures

The plate count, culturing, isolation, identification and antimicrobial susceptibility tests were performed in the microbiology laboratory according to the standard operating procedures of the department. Pour plate count was used for bacterial enumeration, after serial dilution (10^−1^, 10^−2^, 10^−3^) of a neat solution of saline and post-analyzed filter paper. Pour plating was done in triplicate for each dilution factor onto pour plate agar and incubated overnight at 37 °C. Bacteria that grew on pour plate agars were enumerated and sub-cultured onto routine bacteriological media for further isolation and identification. Bacterial isolates were identified using Gram stain, the catalase test and the coagulase test for *Staphylococcus* species, whilst biochemical tests (indole, urease, triple sugar iron) were used to identify Gram-negative isolates. Antimicrobial susceptibility testing and interpretation was performed according to the Clinical and Laboratory Standards Institute (CLSI) guidelines (2020, M30) [16]. In brief, antimicrobial susceptibility testing was performed using the Kirby–Bauer disk diffusion technique where a standardized inoculum (0.5 McFarland) from a pure culture was seeded onto Mueller Hinton Agar. Excess moisture on the agar surface was absorbed prior to application of the antimicrobial disks. The disks were selected for their Gram-positive and Gram-negative bacterial isolates. Plates were incubated aerobically at 35 ± 2 degrees C for 18–24 h, after which zones of inhibition were measured using calipers. The antibiotics that were selected were based on their common use and the identified strains. 

#### 2.5.2. Data Collection Procedures

Data were extracted from the EPA ambient air quality database available in both hardcopy and softcopy from the Environmental Quality Department of the Agency. Data were then exported into Microsoft Excel and kept in a password-protected computer by the principal investigator. 

### 2.6. Data Analysis 

Summary statistics (median and range) were calculated to report on the environmental and metrological characteristics of the sites as well as the airborne bacterial count in each site. The PM_10_ concentration was calculated using a standard formula. The differences in the pre-weighed values of filter paper minus post-weighed values of filter paper were used to calculate the particulate matter in µg/m^3^. The formula below was used:

(1) $${\mathrm{PM}}_{10}\;\left(\frac{\mu g}{m^{3}}\right)=\frac{Net\;dust\;weight\;\left(g\right)*10^{6}}{Flow\;rate\;\left(\frac{L}{\mathrm{Min}}\right)*Sampling\;time\;\left(\mathrm{Min}\right)}$$

Numbers and proportions were calculated to report on the presence and resistance profile of the selected bacteria and their antibiotic resistance profiles for the three types of site. The association between PM_10_ and airborne bacterial counts was presented in a scatter plot and a correlation calculated using Spearman’s correlation coefficient (r_s_). Significance levels were set at 5% using two-tailed significance values.

## 3. Results

### 3.1. Environmental and Meteorological Characteristics and Airborne Bacterial Counts

Environmental and meteorological characteristics of the ambient air sampled from 12 sites are shown in Table 1. Roadside sites had a higher median temperature, a higher median relative humidity and a higher median wind speed compared with industrial sites, which in turn had higher median measurements compared with residential sites. With respect to PM_10_ concentrations, the median level was highest in the roadside sites, while residential sites had a higher median level compared with industrial sites. 

Airborne bacterial counts of the ambient air are shown in Table 2. The median bacterial count was highest in the roadside sites (115,000 CFU/m^3^), followed by the industrial (35,150 CFU/m^3^) and then residential sites (1210 CFU/m^3^). 

### 3.2. Presence of Selected Bacteria in Ambient Air Samples

The presence of the seven selected bacteria in the ambient air is shown in Table 3**.** Diverse and varied bacterial species were obtained from samples collected from roadside, followed by industrial and residential sites. *Bacillus* species were present in the air of all 12 sites. 

### 3.3. Antibiotic Resistance Profiles of the Bacteria Identified in Ambient Air Samples 

Resistance profiles of bacterial isolates from sampled ambient air to four selected antibiotics are shown in Table 4. 

Of the 24 bacterial isolates identified from the 12 sites, six showed resistance to one or more antibiotics. Excluding *Bacillus* species, which was found in every site, there were altogether 12 pathogenic bacteria isolated, of which six (50%) showed mono-resistance or multidrug resistance. 

In terms of the different sites (data not shown), there were 14 bacterial isolates identified from the roadside sites, of which four showed resistance to one or more antibiotics. Of the six bacterial isolates identified from industrial sites, two showed resistance to one or more antibiotics. Of the four bacterial isolates identified from residential sites, none showed resistance to any antibiotic. 

### 3.4. Association between PM_10_ Concentrations and Airborne Bacterial Counts, Selected Bacteria and Antibiotic Resistance 

**Figure 1:**
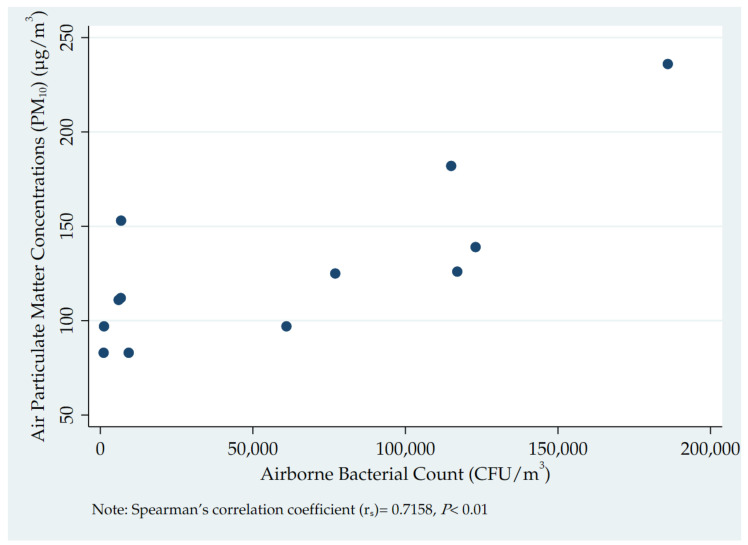
Association between airborne bacterial counts and PM_10_ concentrations at the sites (roadside, industrial and residential) from which ambient air was sampled in Accra, Ghana, February 2020.

Airborne bacterial counts in the 12 sites in relation to PM_10_ concentrations are shown in Figure 1. There was a positive correlation between PM_10_ concentrations and airborne bacterial counts (CFU/m^3^), which was statistically significant (r_s_ = 0.72158, *p* = 0.01). There were no significant correlations between PM_10_ concentrations and presence of selected bacteria or between PM_10_ concentrations and antibiotic resistance patterns (data not shown). 

## 4. Discussion

This is the first study in Ghana to report on the presence and resistance profile of airborne bacteria in the ambient air of roadside, industrial and residential sites, as well as the meteorological and environmental characteristics of these sites. Our study revealed three important findings. 

First, the roadside sites had higher recorded temperatures, humidity, wind speed and PM_10_ concentrations compared with the other two types of sites. Roadside sites also had higher airborne bacterial counts than the other types of sites. We found a strong overall correlation between air pollution (measured as PM_10_ concentrations) and airborne bacterial counts. These findings are not surprising and have been reported by others. For example, a study in an urban area of Pakistan found antibiotic-resistant airborne bacteria in a number of roadside sites in both industrial and residential areas. [17]. Gao et al. (2016) found a strong positive relationship between airborne bacterial concentrations and population density, human activity and road traffic movement, all of which characterize roadside sites [18]. Similarly, in India, high levels of airborne bacteria and particulate matter were found at a high-traffic-density junction in Dehradun city [19]. In Singapore [20], the concentrations of outdoor airborne bacteria were strongly dependent on ambient temperature, and in South Africa, bacterial and fungal concentrations in the air were higher in summer compared with winter [21]. These findings support a possible link between higher temperatures at the roadside sites and their higher airborne bacterial counts. 

Second, we identified all seven of the selected bacteria (*Staphylococcus* species*, Streptococci*, *Escherichia coli*, *Coliforms*, *Pseudomonas aeruginosa*, *Pseudomonas* species and *Bacillus* species*)* at the sample sites. *Bacillus* species are aerobic, sporulating, rod-shaped bacteria that are ubiquitous in nature and widespread in every natural environment [22], and it was encouraging to find that we could identify this particular bacterium from all 12 of our sampling sites, suggesting that our sampling methodology for bacterial culture and drug sensitivity testing was robust. The identification of the other six pathogenic bacteria accords with previous studies conducted elsewhere. Airborne *Staphylococcus aureus* has been identified in residential areas, animal breeding sites and other outdoor public places in several countries [23], and in Bangladesh it was found in three outdoor sites at a university campus [24]. In a market in the middle of a city center in India, the most commonly found Gram-positive cocci was *Staphylococcus aureus*, although Gram-negative bacilli such as *Escherichia coli*, *Pseudomonas* species and *Coliforms* were the overall dominant bacteria isolated [25]. In Nigeria, several bacterial species including *Bacillus* species, *Pseudomonas aeruginosa* and *Coliforms* were isolated from the air in four markets [26]. 

Third, we used four selected antibiotics (penicillin, ampicillin, ciprofloxacin and ceftriaxone) to determine the levels of resistance to bacterial isolates in the ambient air. They were selected because they are four commonly used and widely available antibiotics to treat bacterial infections in humans in Ghana. Excluding the *Bacillus* species, all of which were sensitive to the four antibiotics, six (50%) of the other 12 bacteria which were isolated showed some form of resistance to one or more selected antibiotics. All of the isolated *Escherichia coli* showed multidrug resistance to ampicillin, ciprofloxacin and ceftriaxone, while half of the isolated *Staphylococcal* species showed multidrug resistance to penicillin, ampicillin and ceftriaxone. The isolated *Coliform* bacteria and the isolated non-hemolytic *Streptococci* showed mono-resistance to ceftriaxone and ampicillin, respectively. These findings accord with those published elsewhere, showing high rates of antibiotic resistance to airborne *Staphylococci* species and Gram-negative bacilli [17,27]. The study in indoor sites of a university in Ghana also found high rates of antibiotic resistance in airborne bacteria that included both Gram-positive cocci and Gram-negative bacilli [10]. 

The strengths of this study are that we used all active air sample sites in urban Accra. The isolation of *Bacillus* species in all our cultured samples reassured us that our sampling and microbiological procedures were in order. We contributed to the WHO AMR surveillance report by considering six pathogenic bacteria in total, which are part of the priority list provided by WHO [28]. We also looked at four different antibiotics, which are the most important antibiotics used in Ghana to treat bacterial infections in adults and children. The conduct and reporting of this study adhered to the STROBE (Strengthening the Reporting of Observational Studies in Epidemiology) guidelines [29]. 

There were, however, some limitations. We only took one sample of ambient air from each site and it might have been better to take several samples over a defined period. In addition, taking one sample from each site meant that our sample size was small. We also did not collect and analyze ambient air samples at different seasons of the year. Other studies have found seasonal variation in the bacterial count and antibiotic resistance profiles [17,19,20]. We had planned to do sample collection at different times from 2020 onwards and in different seasons. However, the COVID-19 pandemic restricted movement in the city and made these plans impossible to implement. Further research is, therefore, needed to understand the presence and resistance pattern of bacteria in the ambient air of urban Accra in different seasons. We did not measure PM_2.5_ (which is a measure of fine particulate matter in the air) as the machine components for this were not available at the time of the study. Finally, at the time of the study we were unable to identify some of the bacterial isolates down to their species level because of inherent limitations in the identification methods used. 

Despite the limitations, there are some important programmatic implications of this study. First, there is a recent and growing interest in countries to monitor ambient air, not only for environmental and meteorological characteristics but also for identifying antibiotic-resistant bacteria, and Ghana has joined that list. The EPA in Ghana now needs to use these 12 sites in urban Accra to regularly monitor for antibiotic-resistant bacteria. It also needs to consider upscaling by setting up further air sampling sites in other cities in the country in order to have country-wide and nationally representative data. Ghana has good policy and regulatory systems to assess antibiotic use and resistance in the country [30], and regular data and information from ambient air samples can add to the evidence base. 

Second, more work needs to be done to establish the importance of ambient outdoor airborne antibiotic-resistant bacteria with respect to AMR in humans and animals. Recent studies in Ghana have established a link between household air pollution and bacterial nasal carriage in infants [31] and have also shown that exposure to smoke in the air is a risk factor for meningococcal meningitis [32]. In China, studies have shown that urban air is being gradually polluted by bacterial antibiotic resistance genes and this resistance gene pollution has been increasing over the last ten years [33]. This suggests that urban airborne transmission of antibiotic resistance genes is a potential public health threat and emphasizes the need to improve air quality standards. It also endorses the need for a “one health approach” that involves many other sectors, such as agriculture and human and animal health.

Third, Ghana needs to continue with this important work on regularly measuring and reporting on airborne bacterial pollution and the associated antibiotic resistance in its urban cities. It should also consider expanding this work to other outdoor areas likely to be hotspots, and take the appropriate actions not only to improve the health of its population but to contribute to clean and thus resilient cities.

Finally, further research is needed to investigate the sources of antibiotic-resistant bacteria in the ambient air of Accra and elsewhere in Ghana. 

## 5. Conclusions

Although the findings of this study on ambient air in urban Accra from roadside, industrial and residential sites are preliminary, the study has provided a snap shot of the presence and resistance profiles of selected airborne bacteria. It has shown that roadside sites have higher rates of adverse environmental, meteorological and airborne bacterial concentrations compared with the other sites. Pathogenic bacteria can be found in all types of sites with half of these bacteria exhibiting mono-resistance or multidrug resistance to antibiotics. Recommendations for further action have been made. The study confirms the importance of antimicrobial resistance as an urgent and important global public health problem, and shows that antibiotic drug susceptibility/resistance testing and antibiotic stewardship are critical components of guidelines and institutional and health worker prescribing practices. 

## Figures and Tables

**Table 1:** Environmental and meteorological characteristics for the three types of site (roadside, industrial and residential) from which ambient air was sampled in urban Accra, Ghana (February 2020).

	Sampling Sites N	Temperature (°C) Median (Range)		Relative Humidity (%) Median (Range)		Wind Speed (km/h) Median (Range)		PM_10_ Concentration (µg/m^3^) Median (Range)	
All sites	12	32	(27–38)	84	(66–96)	21	(16–25)	119	(83–236)
Roadside	7	35	(31–38)	86	(66–96)	21	(18–25)	126	(97–236)
Industrial	2	29	(29–30)	84	(82–86)	19	(16–22)	90	(83–97)
Residential	3	27	(27–38)	80	(79–82)	18	(16–20)	111	(83–153)

N: number of sites, PM_10_: particulate matter with effective size of less than 10 microns.

**Table 2:** Airborne bacteria count for the three types of site (roadside, industrial, residential) from which ambient air was sampled in urban Accra, Ghana (February 2020).

	Sampling Sites N	Airborne Bacteria Count (CFU/m^3^) Median (Range)	
All sites	12	35,150	(1080–186,000)
Roadside	7	115,000	(6700–186,000)
Industrial	2	35,150	(9300–61,000)
Residential	3	1210	(1080–6000)

N: number of sites, CFU/m^3^: colony-forming units per cubic meter.

**Table 3:** Bacteria isolated from post-analyzed ambient air filter papers sampled from three sites (roadside, industrial, residential) in urban Accra, Ghana (February 2020).

Organisms	Type of Site					
	Roadside n = 7		Industrial n = 2		Residential n = 3	
		(%)		(%)		(%)
*Pseudomonas aeruginosa*	0	(0)	0	(0)	1	(33)
*Escherichia coli*	1	(14)	1	(50)	0	(0)
*Pseudomonas* species	2	(29)	1	(50)	0	(0)
Non-hemolytic *Streptococci*	1	(14)	0	(0)	0	(0)
*Bacillus* species	7	(100)	2	(100)	3	(100)
*Coliforms*	1	(14)	0	(0)	0	(0)
*Staphylococci* species	2	(29)	2	(50)	0	(0)

n = number of sites from which ambient air was sampled; % = column percentages based on number of organisms in each site.

**Table 4:** Resistance profiles of bacterial isolates to selected antibiotics for the sites from which ambient air was sampled in urban Accra, Ghana (February 2020).

Bacterial Isolates	Resistance Profiles to Selected Antibiotics	N	(%)
*Pseudomonas aeruginosa*		1	
	Penicillin	0	
	Ampicillin	0	
	Ciprofloxacin	0	
	Ceftriaxone	0	
*Escherichia coli*		2	
	Penicillin	0	
	Ampicillin	2	(100)
	Ciprofloxacin	2	(100)
	Ceftriaxone	2	(100)
*Pseudomonas* species		3	
	Penicillin	0	
	Ampicillin	0	
	Ciprofloxacin	0	
	Ceftriaxone	0	
Non-hemolytic *Streptococci*		1	
	Penicillin	0	
	Ampicillin	1	(50)
	Ciprofloxacin	0	
	Ceftriaxone	0	
*Bacillus* species		12	
	Penicillin	0	
	Ampicillin	0	
	Ciprofloxacin	0	
	Ceftriaxone	0	
*Coliforms*		1	
	Penicillin	0	
	Ampicillin	0	
	Ciprofloxacin	0	
	Ceftriaxone	1	(50)
*Staphylococci* species		4	
	Penicillin	2	(50)
	Ampicillin	2	(50)
	Ciprofloxacin	0	(50)
	Ceftriaxone	2	(50)

% = column percentages showing the proportion of bacterial isolates resistant to one or more of the selected antibiotics.

## Data Availability

The data that support the findings of this study are available from the corresponding author G.S.K.A. upon a reasonable request.

## Supplementary Materials

The following are available online at https://www.mdpi.com/article/10.3390/tropicalmed6030110/s1, Annex S1: Description of the 12 sites from which ambient air samples were obtained in urban Accra, Ghana, February 2020.

## References

[1] World Health Organisation (2019). The Journal of Global Antimicrobial Resistance meets the World Health Organization (WHO). J. Glob. Antimicrob. Resist..

[2] Kuralayanapalya S.P., Patil S.S., Hamsapriya S., Shinduja R., Roy P., Id R.G.A. (2019). Prevalence of extended-spectrum beta- lactamase producing bacteria from animal origin: A systematic review and meta-analysis report from India. PLoS ONE.

[3] Ahmad Z., Colbeck I., Sultan S., Ahmed S. (2012). Bioaerosols in residential micro-environments in low income countries: A case study from Pakistan. Environ. Pollut..

[4] Nilsson A., Kihlström E., Lagesson V., Wessen B., Szponar B., Larsson L., Tagesson C. (2004). Microorganisms and volatile organic compounds in airborne dust from damp residences. Indoor Air.

[5] Bragoszewska E., Mainka A., Pastuszka J.G., Lizonczyk K., Desta Y.G. (2018). Assessment of Bacterial Aerosol in a Preschool, Primary School and High School in Poland. Atmosphere.

[6] Brochu C.A. (2006). Eosuchus (Crocodylia, Gavialoidea) from the lower Eocene of the Isle of Sheepey, England. J. Vertebr. Paleontol..

[7] De Freitas L.C. (2013). Global Prio. Cad. Pesqui..

[8] Lee T., Grinshpun S.A., Martuzevicius D., Adhikari A., Crawford C.M., Reponen T. (2006). Culturability and concentration of indoor and outdoor airborne fungi in six single-family homes. Atmos. Environ..

[9] Pompilio A., Di Bonaventura G. (2020). Ambient air pollution and respiratory bacterial infections, a troubling association: Epidemiology, underlying mechanisms, and future challenges. Crit. Rev. Microbiol..

[10] Abiola I., Abass A., Duodu S., Mosi L. (2018). Characterization of culturable airborne bacteria and antibiotic susceptibility profiles of indoor and immediate-outdoor environments of a research institute in Ghana. AAS Open Res..

[11] Mansour G., Esseku H. (2017). Situation Analysis of the Urban Sanitation Sector in Ghana.

[12] EPA (2018). The Greater Accra Metropolitan Areas Air Quality Management Plan 2018.

[13] GSA (2018). Ghana Standard for Environment and Health Protection, Requirements for Motor Vehicle Emissions.

[14] Breu F., Guggenbichler S., Wollmann J. Environmental Protection Agency Act, 1994 Act 490. http://medcontent.metapress.com/index/A65RM03P4874243N.pdf.

[15] GSA (2019). Ghana Standard for Environment and Health Protection-Requirements for Ambient Air Quality.

[16] Hudzicki J. (2009). Kirby-Bauer Disk Diffusion Susceptibility Test Protocol Author Information. https://www.asm.org/Protocols/Kirby-Bauer-Disk-Diffusion-Susceptibility-Test-Pro.

[17] Naz N., Nasim F.U.H., Pasha T.S. (2019). Prevalence of antibiotic-resistant airborne bacteria along roadsides in Rahim Yar Khan, Pakistan. Pol. J. Environ. Stud..

[18] Gao X.L., Shao M.F., Luo Y., Dong Y.F., Ouyang F., Dong W.Y., Li J. (2016). Airborne bacterial contaminations in typical Chinese wet market with live poultry trade. Sci. Total Environ..

[19] Madhwal S., Prabhu V., Sundriyal S., Shridhar V. (2020). Ambient bioaerosol distribution and associated health risks at a high traffic density junction at Dehradun city, India. Environ. Monit. Assess..

[20] Rajasekar A., Balasubramanian R. (2011). Assessment of airborne bacteria and fungi in food courts. Build. Environ..

[21] Morakinyo O.M., Mokgobu M.I., Mukhola M.S. (2019). Biological Composition of Respirable Particulate Matter in an Industrial Vicinity in South Africa. Int. J. Environ. Res. Public Health.

[22] Turnbull P.C.B., Baron S. (1996). Bacillus. Galveston (TX).

[23] Kozajda A., Jeżak K., Kapsa A. (2019). Airborne Staphylococcus aureus in different environments—A review. Environ. Sci. Pollut. Res..

[24] Kabir M.S., Mridha F., Islam S., Shorifujjaman M. (2016). Microbiological pollutants in air and antibiotic resistance profile of some bacterial isolates. Jahangirnagar Univ. J. Biol. Sci..

[25] Naruka K., Gaur J. (2014). Distribution Pattern of Airborne Bacteria and Fungi at Market Area. Am. J. Sci. Res..

[26] Ogugbue C. (2011). Assessment of microbial air contamination of post processed garri on sale in markets. Afr. J. Food Sci..

[27] Islam A., Kabir S., Khair A. (2019). Molecular Identification and Evaluation of Indigenous Bacterial Isolates for Their Plant Growth Promoting and Biological Control Activities against Fusarium Wilt Pathogen of Tomato. Plant Pathol. J..

[28] World Health Organisation (WHO) (2014). Antimicrobial Resistance: Global Report on Surveillance 2014.

[29] Von Elm E., Altman D.G., Egger M., Pocock S.J., Gøtzsche P.C., Vandenbroucke J.P. (2014). The Strengthening the Reporting of Observational Studies in Epidemiology (STROBE) Statement: Guidelines for reporting observational studies. Int. J. Surg..

[30] Yevutsey S.K., Buabeng K.O., Aikins M., Anto B.P., Biritwum R.B., Frimodt-Møller N., Gyansa-Lutterodt M. (2017). Situational analysis of antibiotic use and resistance in Ghana: Policy and regulation. BMC Public Health.

[31] Carrión D., Kaali S., Kinney P.L., Owusu-Agyei S., Chillrud S., Yawson A.K., Quinn A., Wylie B., Ae-Ngibise K., Lee A.G. (2019). Examining the relationship between household air pollution and infant microbial nasal carriage in a Ghanaian cohort. Environ. Int..

[32] Hodgson A., Smith T., Gagneux S., Adjuik M., Pluschke G., Mensah N.K., Binka F., Genton B. (2001). Risk factors for meningococcal meningitis in northern Ghana. Trans. R. Soc. Trop. Med. Hyg..

[33] Li Y., Liao H., Yao H. (2019). Prevalence of antibiotic resistance genes in air-conditioning systems in hospitals, farms, and residences. Int. J. Environ. Res. Public Health.

