# Supplementary material for: Bacteria and Their Antibiotic Resistance Profiles in Ambient Air in Accra, Ghana, February 2020: A Cross-Sectional Study
Source: Trop Med Infect Dis. 2021 Jun 25;6(3):110. doi: 10.3390/tropicalmed6030110 (PMC8293412; doi:10.3390/tropicalmed6030110)
Supplement: Supplementary file 1 [file tropicalmed-06-00110-s001.zip › tropicalmed-1191277-supplementary.pdf]

Supplementary Annex to main paper.

Description of the 12 sites from which ambient air samples were obtained in urban Accra, Ghana, February 2020.

Site type	Site location	Site description	
Industrial	E - South industrial area	Located close to the State Transport Company yard in the south industrial area. The area is also close to river Odaw and the Agbogbloshie scrap metal area.	P
	D - North industrial area	Located close to North industrial area Ghana Commercial Bank and Latex Foam manufacturing company. The area is also close to Bubuashie in the North industrial area. There are scattered residential facilities in the area.	P
Residential	A - East Legon, Friends of the earth area	Located in the premises of Friends of the Earth, an environmental non-governmental organization (NGO) in the area. The site is about 60 meters (m) away from the Accra - Medina main road and about 20 m away from the East Legon main drain. Emmanuel Eye Specialist Clinic is about 100 m away from the site. East Legon Shashie area is a predominantly high-class residential area in Accra.	P
	B - Dansoman Police Station	The site is located in predominantly residential facilities in the Ablekuma west municipality.	P
	G - Odorkor SDA School	Located close to Odorkor SDA School area. The area includes both residential facilities and a commercial area.	P
Roadside	1 - Kaneshie first light	Located on the pedestrian’s refuge island of the Accra – Kasoa main road. The area is predominantly surrounded by residential facilities and market activities. It is also one of the vehicle traffic prone areas in the city. Street hawkers and other pedestrians are often found in the area during the daytime.	
	3 - Achimota Interchange area	Located in the interchange between the Tetteh Quashie to Mallam road corridor and Accra – Nsawam road. The site is also close to some scattered residential and	

		commercial facilities in the area. Street hawkers and other pedestrians are often seen in the area.	
	2 - Tetteh Quashie Interchange Aviation house area	Located in the pedestrian's refuge island of the Accra – Tema/Medina road and in the Airport residential enclave. Vehicle traffic in the area is always very heavy with street hawking activities.	
	5 - Mallam Junction market area	Located opposite the Mallam market off the Accra – Kasoa road. The site is surrounded by scattered residential and commercial facilities in the area. It is also several meters away from the Mallam junction lagoon. Vehicle traffic and street hawking in the area is always very heavy.	
	8 – Kasoa, Galilee market area	Located opposite to the Kasoa Galilee market. Pedestrian footpaths and fuel filling stations are located close to the site. There are a few residential facilities around the site.	
	10 - Amasaman Fise Junction	Located in the premises of Puma Energy (fuel filling station). There is a taxi station located about 20 m adjacent to the monitoring site. The monitoring site is also off the Pokuase – Nsawam road in the Ga West Municipality. The site is surrounded by several residential units and some commercial facilities.	
	9 - Tantra Hill, St. Johns Grammar area	Located in the premises of Goil fuel filling station in the Ga West Municipality. The site is about 10 m away from the Achimota – Pokuase main road. The area is purely residential.	

EPA: Environmental Protection Agency; P: permanent site; SDA: Seventh-Day Adventist;
